# Transcriptomic Insights into Functions of *LkABCG36* and *LkABCG40* in *Nicotiana tabacum*

**DOI:** 10.3390/plants12020227

**Published:** 2023-01-04

**Authors:** Nan Sun, Can Li, Xiangning Jiang, Ying Gai

**Affiliations:** 1 College of Biological Sciences and Biotechnology, Beijing Forestry University, Beijing 100083, China; 2 State Key Laboratory of Tree Genetics and Breeding, College of Biological Sciences and Technology, Beijing Forestry University, Beijing 100083, China; 3 National Engineering Laboratory for Tree Breeding, The Tree and Ornamental Plant Breeding and Biotechnology Laboratory of Chinese Forestry Administration, Beijing 100083, China

**Keywords:** ABC transporter, *LkABCG36*, *LkABCG40*, transcriptomic

## Abstract

ATP-binding cassette transporters (ABC transporters) play crucial physiological roles in plants, such as being involved in the growth and development of organs, nutrient acquisition, response to biotic and abiotic stress, disease resistance, and the interaction of the plant with its environment. The ABCG subfamily of proteins are involved in the process of plant vegetative organ development. In contrast, the functions of the ABCG36 and ABCG40 transporters have received considerably less attention. Here, we investigated changes in the transcriptomic data of the stem tissue of transgenic tobacco (*Nicotiana tabacum*) with *LkABCG36* and *LkABCG40* (*Larix kaempferi*) overexpression, and compared them with those of the wild type (WT). Compared with the WT, we identified 1120 and 318 differentially expressed genes (DEGs) in the *LkABCG36* and *LkABCG40* groups, respectively. We then annotated the function of the DEGs against the Gene Ontology (GO) and Kyoto Encyclopedia of Genes and Genomes (KEGG) databases. The results showed enrichment in cell wall biogenesis and hormone signal transduction functional classes in transgenic *LkABCG36* tobacco. In transgenic *LkABCG40* tobacco, the enrichment was involved in metabolic and biosynthetic processes, mainly those related to environmental adaptation. In addition, among these DEGs, many auxin-related genes were significantly upregulated in the *LkABCG36* group, and we found key genes involved in environmental adaptation in the *LkABCG40* group, including an encoding resistance protein and a WRKY transcription factor. These results suggest that *LkABCG36* and *LkABCG40* play important roles in plant development and environmental adaptation. *LkABCG36* may promote plant organ growth and development by increasing auxin transport, whereas *LkABCG40* may inhibit the expression of *WRKY* to improve the resistance of transgenic tobacco. Our results are beneficial to researchers pursuing further study of the functions of ABCG36 and ABCG40.

## 1. Introduction

ATP-binding cassette transporters (ABC transporters) are membrane-associated primary transporters forming a large and ubiquitous family that plays important roles in the physiological development of plants [1], including the growth and development of organs, nutrient acquisition, response to abiotic and biotic stress, disease resistance, and the interaction of the plant with its environment [2,3,4]. ABC transporters are mainly localized in the plasma membrane, tonoplast, chloroplast, mitochondria, and peroxisomes on the membrane structures of plants [5,6]. The spectrum of substrates that can be transported by ABC transporters ranges from small molecules such as heavy metals, inorganic acids, and peptides, to large molecules such as lipids, polysaccharides, steroids, and even intact proteins [7,8]. Compared with other eukaryotes, plants have more abundant ABC transporters, especially the ABCG subgroup [9,10].

ABCG transporters are involved in diverse processes in many plant species, including pathogen response, diffusion barrier formation, response to abiotic stress, and phytohormone transport [11,12]. As such, the dysfunction of some ABCG transporters profoundly affects plant growth, resulting in partial or complete sterility [13]. For example, AtABCG26 (*Arabidopsis*) mainly transports polyketides for antioxidant capacity; loss of function of AtABCG26 leads to sterility and reduced fertility [14]. Furthermore, ABCG transporters are involved in transporting lipid molecules, such as wax and keratin, to the epidermis to promote the stress resistance of the plant [15]. NpABCG5 (*Nicotiana plumbaginifolia*) is involved in facilitating a plant’s resistance to pathogens by transporting lipid molecules [16]. The ABCG transporters also play a crucial role in phytohormone transport over long distances in various directions [10]. For example, AtABCG1 and AtABCG16 can change the distribution and flow of auxin by transporting growth hormone that ensure the rapid growth of pollen tubes in pistils [17,18]. AtABCG37 transports substrates involved in auxin synthesis, such as endogenous auxin precursor indole-3-butyrate (IBA), which plays an important role in plant growth and development [19].

The AtABCG36 transporter plays a dual role in the transport of IBA [20,21], and AtABCG40 is a high-affinity abscisic acid (ABA) transporter [22]. However, no studies have been conducted on the function of *LkABCG36* and *LkABCG40* in *Larix kaempferi.* In addition, the mechanisms of the effects of *LkABCG36* and *LkABCG40* on plant organ growth, development, and environmental adaptation are still unclear. As such, to explore the function of *LkABCG36* and *LkABCG40*, we overexpressed these genes in a model organism (*Nicotiana tabacum*). 

Gene expression analysis is useful for studying the molecular mechanisms and physiology of plants. Many researchers have identified interesting marker genes with advantageous traits or the molecular mechanisms of target genes based on the differences in the mRNA expression profiles between wild type and transgenic groups.

In this study, we performed a transcriptomic analysis on the stem tissues from transgenic tobacco overexpressing *LkABCG36* and *LkABCG40,* and we compared the results with those of wild type tobacco. We first searched for significant DEGs, and then performed GO and KEGG functional enrichment analysis on the DEGs. We screened a series of marker genes that may play crucial roles in the improvement of transgenic plant traits. Our findings provide insights into the functions of ABCG transporters in plant development and elucidate the molecular mechanisms of *LkABCG36* and *LkABCG40*.

## 2. Results

### 2.1. Overexpression of LkABCG36 and LkABCG40 Promotes Plant Growth in Tobacco

**Figure 1:**
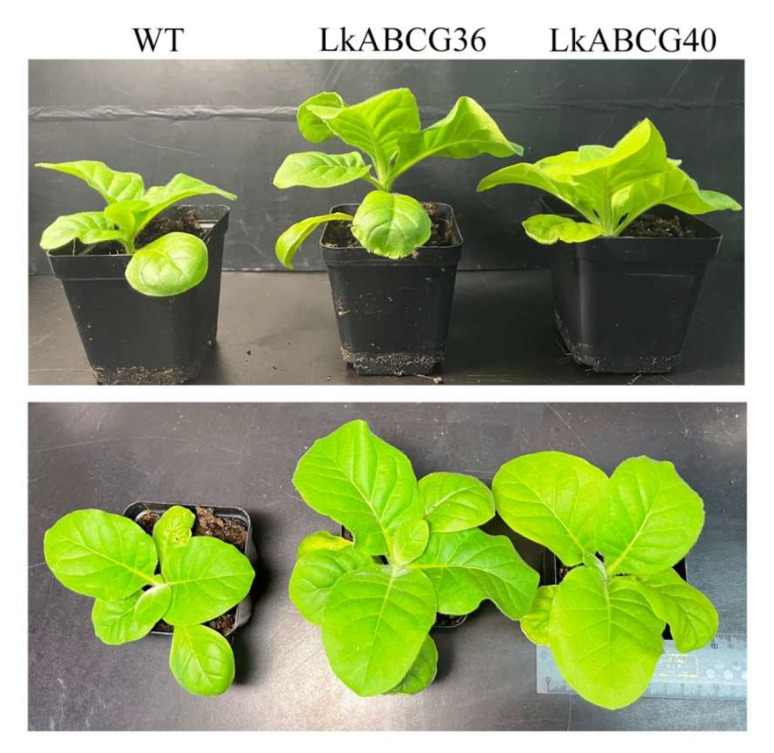
Overexpression of *LkABCG36* and *LkABCG40* promoted tobacco plant growth. Morphology of wild type (WT) and transgenic (*LkABCG36* and *LkABCG40*) tobacco plants at 11 weeks old.

To evaluate the effect of the overexpression of *LkABCG36* and *LkABCG40* on tobacco growth, we first examined the growth behavior and phenotype of these lines. Compared with the wild type (WT), the transgenic tobacco (*LkABCG36* and *LkABCG40*) had larger leaves and longer stems under the same growth conditions (Figure 1). Next, we obtained transgenic plant stem tissues for use in transcriptome analysis to explore the functions of *LkABCG36* and *LkABCG40* and their effects on transgenic tobacco growth.

### 2.2. Transcriptome Analysis in Stem of Transgenic Tobacco Plants Overexpressing LkABCG36 and LkABCG40

**Figure 2:**
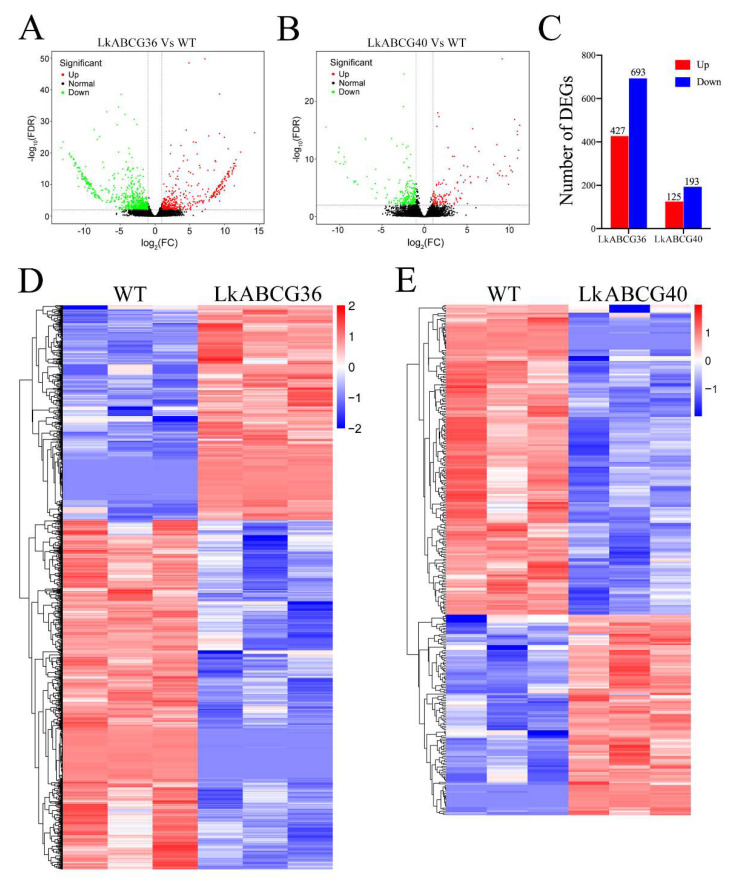
Transcript level profiles analysis of significantly differentially expressed genes. (**A**,**B**) Volcano plots of significantly differentially expressed genes (DEGs) between *LkABCG36* (**A**) or *LkABCG40* (**B**) and WT groups. DEGs were identified by comparing gene expression values with |log_2_(fold change)| ≥ 1 and *p* < 0.05. (**C**) Number of DEGs was counted. We found 427 and 693 significantly up- and downregulated genes, respectively, in *LkABCG36* group compared with WT group. We found 125 and 193 significantly up- and downregulated genes, respectively, in *LkABCG40* group compared with WT group. (**D**,**E**) Heatmap of DEGs between ABCG36 and WT groups (**D**), and between *LkABCG40* and WT groups (**E**). FPKM values (transcript levels) were transformed to log_10_ (FPKM) for color scaling.

We first screened the DEGs according to the two classic criteria of |log_2_ (FC)| > 1 and significance level *p* < 0.05. The volcano plots of the significant DEGs between the *LkABCG36* and WT groups are shown in Figure 2A,C. We identified 427 and 693 significantly up- and downregulated DEGs, respectively, in the *LkABCG36* group compared with the WT group. The volcano plots of the significant DEGs between the *LkABCG40* and WT groups are shown in Figure 2B,C. We identified 125 and 193 significantly up- and downregulated DEGs, respectively, in the *LkABCG40* group compared with WT group. The expression levels of the DEGs are shown as heatmaps in Figure 2D,E.

**Figure 3:**
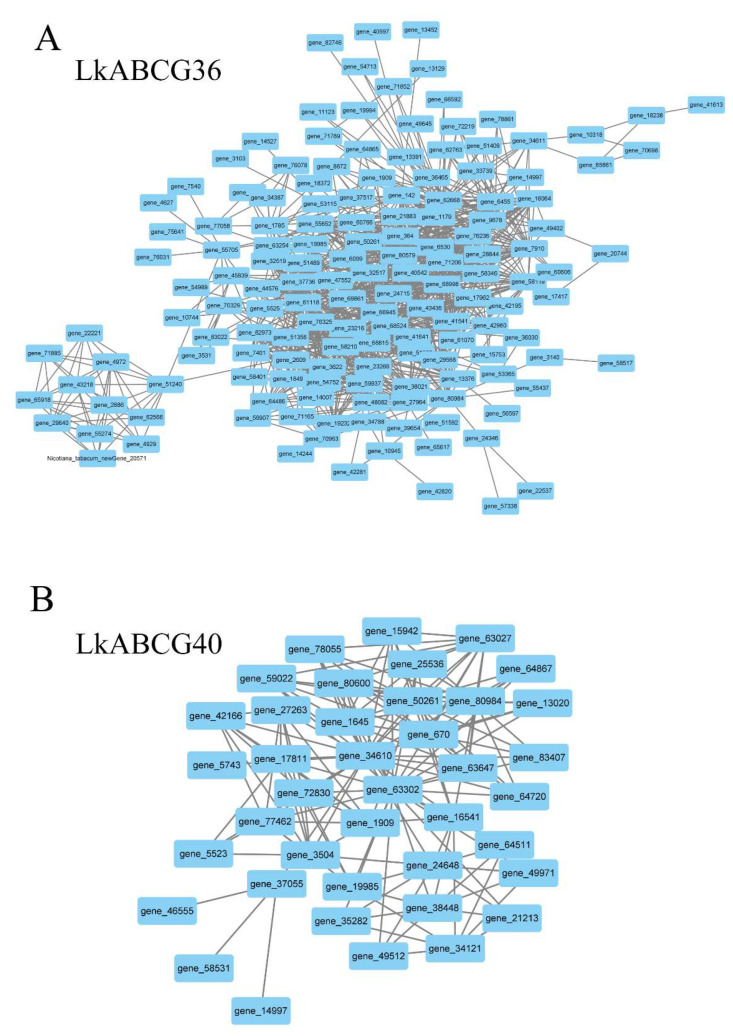
Characterization of DEGs in *LkABCG36* and *LkABCG40* groups. (**A**) Co-expression network analysis of DEGs in *LkABCG36* group; (**B**) Co-expression network analysis of DEGs in *LkABCG40* group.

In addition, to identify the highly correlated DEGs, we characterized the DEGs in the *LkABCG36* and *LkABCG40* groups by co-expression networks analysis. We found that most of the DEGs in the *LkABCG36* group had high correlation coefficients (Figure 3A), whereas fewer DEGs had high correlation coefficients in the *LkABCG40* group (Figure 3B).

### 2.3. Functional Enrichment Analysis of DEGs in Transgenic Tobacco Overexpressing LkABCG36

**Figure 4:**
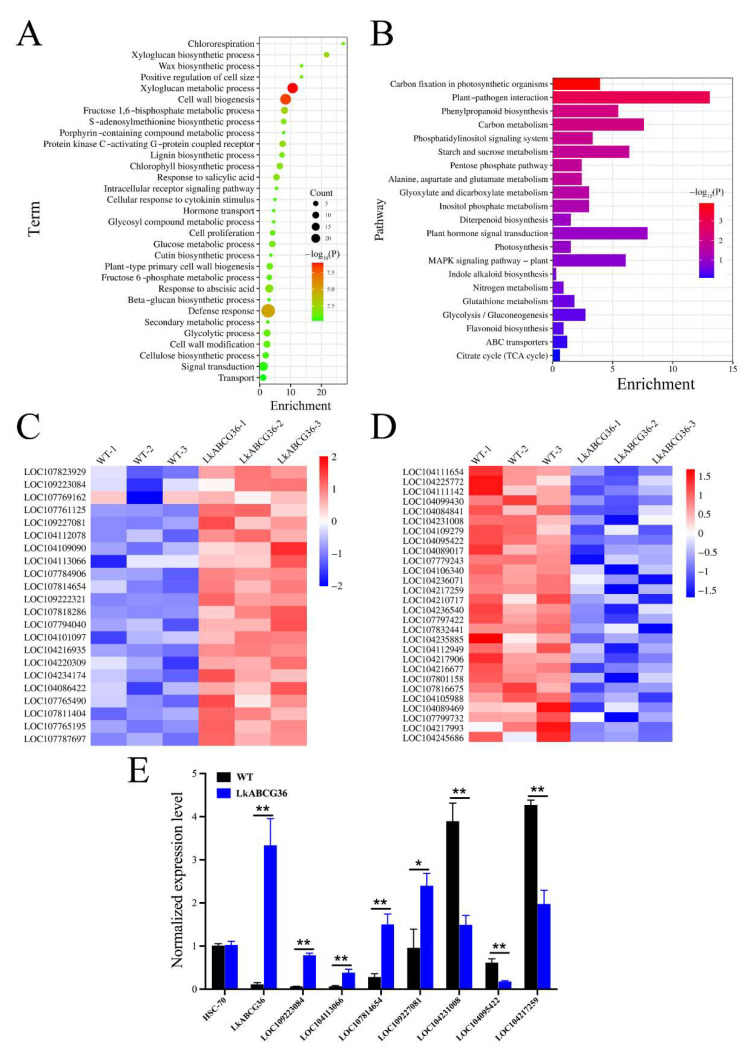
Functional enrichment analysis of DEGs and expression of key genes in *LkABCG36* group. (**A**) Gene Ontology (GO) enrichment analysis identifiers in cluster of overlapping DEGs in *LkABCG36* group compared with WT group. (**B**) Kyoto Encyclopedia of Genes and Genomes (KEGG) pathway enrichment analysis identifiers in cluster of overlapping DEGs in *LkABCG36* group compared with WT group. (**C**) Heatmap shows key upregulated DEGs involved in biosynthesis of some secondary metabolites, energy metabolism, and plant hormone signal transduction. Relative expression level of genes was calculated using log10 (FPKM). (**D**) Heatmap shows key downregulated DEGs involved in biosynthesis of signal transduction and energy metabolism. Relative expression level of genes was calculated using log10 (FPKM). (**E**) We used RT-PCR to detect changes in key up- and downregulated DEGs in stem tissue of transgenic tobacco. Data reported as mean ± SEM. Significance levels are noted as * *p* < 0.05; ** *p* < 0.01.

To explore the functional significance of the genes that were differentially expressed between the *LkABCG36* and WT groups, we first performed Gene Ontology (GO) enrichment analysis (Figure 4A). We found significant enrichment of genes relevant to cell wall biogenesis, including those involved in xyloglucan metabolism, xyloglucan and lignin biosynthetic processes, and cell wall modification. In addition, we found enrichment in energy metabolism processes such as the fructose 1,6-bisphosphate and glucose metabolic processes, the glycolytic process, and chlororespiration. Furthermore, we identified enrichment in the defense response processes, such as in the response to abscisic acid and salicylic acid, the wax biosynthetic process, and the cellular response to cytokinin stimulus. Moreover, certain transport and signal transduction terms were also enriched, such as in hormone transport and intracellular receptor signaling pathways.

To further explore which signal pathways were directly affected by *LkABCG36* overexpression, we analyzed DEG enrichment according to the KEGG pathway database. The results showed that the four main pathways involved were energy metabolism, biosynthesis of other secondary metabolites, environmental information processing, and carbohydrate metabolism (Figure 4B). 

Various pathways, including carbon fixation in photosynthetic organisms, photosynthesis, and nitrogen metabolism, are involved in energy metabolism. In addition, pathways including phenylpropanoid biosynthesis, inositol phosphate metabolism, and flavonoid biosynthesis are involved in the biosynthesis of other secondary metabolites. Pathways including ABC transporters, MAPK signaling pathway—plant, plant hormone signal transduction, and phosphatidylinositol signaling systems are involved in environmental information processing. Finally, pathways including the citrate cycle (TCA cycle), glycolysis/gluconeogenesis, glyoxylate and dicarboxylate metabolism, and starch and sucrose metabolism are involved in carbohydrate metabolism.

To validate the signaling pathways identified in the aforementioned results, as well as to explore the key genes involved in transgenic tobacco overexpressing *LkABCG36*, we studied a number of key genes, and their expression levels are shown in Figure 4C,D. Of these DEGs, many auxin-related genes were significantly upregulated; examples include *LOC109223084*, encoding auxin-responsive protein; *LOC104113066* and *LOC107784906*, encoding indole-3-acetic acid-amido synthetase; and *LOC109227081*, encoding auxin response factor. In addition, genes regulating hormone-related kinases were also significantly upregulated, including *LOC107814654*, *LOC104086422,* and *LOC107761125* (Figure 4C). To further verify the reliability of sequencing, we detected the expression levels of the above genes in the stem of transgenic tobacco using qRT-PCR (Figure 4E). The significantly downregulated DEGs were mainly involved in carbohydrate metabolism; for example, *LOC104231008*, encoding glucose-6-phosphate 1-dehydrogenase; *LOC104109279*, encoding malate dehydrogenase; *LOC104095422* and *LOC104089017*, encoding fructose-bisphosphate aldolase 1; *LOC104217259* and *LOC104112949*, encoding glutamate dehydrogenase B; and *LOC107801158*, encoding glyceraldehyde-3-phosphate dehydrogenase B (Figure 4D). We also used qRT-PCR to detect the expression levels of the above downregulated genes in the stem of transgenic tobacco (Figure 4E). These findings suggested that *LkABCG36* possibly has functions in processes involving both the transport of plant hormones and signal transduction.

### 2.4. Functional Enrichment Analysis of DEGs in Transgenic Tobacco Overexpressing LkABCG40

**Figure 5:**
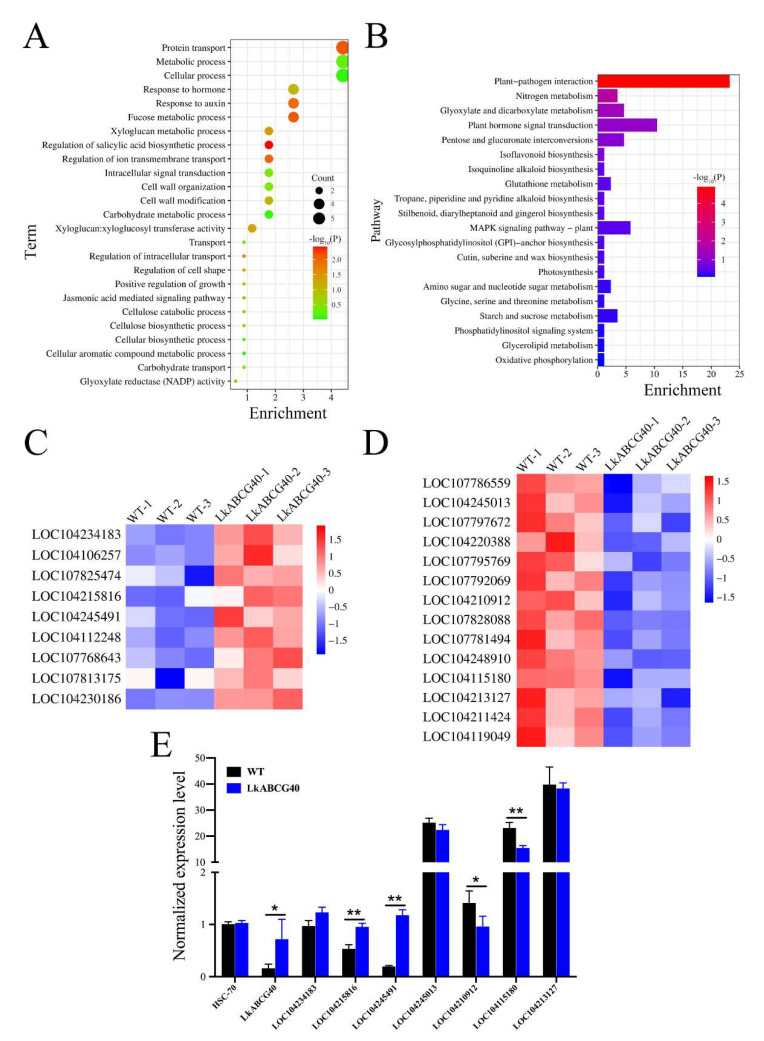
Functional enrichment analysis of DEGs and expression of key genes in *LkABCG40* group. (**A**) Gene Ontology (GO) enrichment analysis identifiers in cluster of overlapping DEGs in *LkABCG40* group compared with WT group. (**B**) Kyoto Encyclopedia of Genes and Genomes (KEGG) pathway enrichment analysis identifiers in cluster of overlapping DEGs in *LkABCG40* group compared with WT group. (**C**) Heatmap shows key upregulated DEGs involved in biosynthesis of environmental adaptation and energy metabolism. Relative expression level of genes was calculated using log_10_(FPKM). (**D**) Heatmap showing the key downregulated DEGs involved in biosynthesis of signal transduction and energy metabolism. Relative expression level of genes was calculated using log_10_ (FPKM). (**E**) RT-PCR was used to detect changes in key up- and downregulated DEGs in stem tissue of transgenic tobacco. Data shown as mean ± SEM. Significance levels are noted as * *p* < 0.05; ** *p* < 0.01.

To explore the functional significance of the DEGs between the *LkABCG40* and WT groups, we performed GO enrichment analysis (Figure 5A). We found that the genes are involved in the regulation of intracellular transport; we noted strong enrichment in protein transport, the regulation of ion transmembrane transport, and carbohydrate transport. In addition, we found gene enrichment in metabolic and biosynthetic processes, such as the salicylic acid and cellulose biosynthetic processes, and the carbohydrate and cellular aromatic compound metabolic processes. Furthermore, the terms including the response to hormones, the jasmonic-acid-mediated signaling pathway, and intracellular signal transduction were also enriched.

To further explore which signal pathways were directly affected by the overexpression of *LkABCG40*, we analyzed DEG enrichment according the KEGG pathway database (Figure 5B). We found that the pathways were mainly involved in four areas: environmental adaptation, energy metabolism, biosynthesis of other secondary metabolites, and signal transduction. 

Pathways including plant–pathogen interaction, and cutin, suberine, and wax biosyntheses are involved in environmental adaptation. In addition, pathways including oxidative phosphorylation, photosynthesis, and nitrogen metabolism are involved in energy metabolism. Furthermore, various pathways, including the biosynthesis of isoflavonoids; of tropane, piperidine, and pyridine alkaloids; and of stilbenoid, diarylheptanoid, and gingerol, are involved in the biosynthesis of other secondary metabolites. Moreover, certain pathways, including plant hormone signal transduction and the MAPK signaling pathway—plant, are involved in signal transduction.

To validate the signaling pathways identified in the aforementioned results, as well as to explore the key genes in transgenic tobacco overexpressing *LkABCG40*, we studied a number of key genes, and their expression levels are shown in Figure 5C,D. Of these DEGs, many key genes involved in environmental adaptation were significantly upregulated, such as *LOC104234183*, encoding probable disease resistance protein RF9; *LOC104215816*, encoding resistance protein homolog R1A-10; and *LOC104245491*, encoding resistance protein homolog R1B-16 (Figure 5C). To further verify the reliability of the sequencing, we detected the expression levels of the above genes in the stem of transgenic tobacco using qRT-PCR (Figure 5E). The significantly downregulated DEGs were mainly involved in plant–pathogen interactions, such as *LOC104245013*, encoding WRKY transcription factor 41; *LOC104210912,* encoding WRKY transcription factor 22; *LOC104115180*, encoding WRKY transcription factor 41-like; *LOC104213127*, encoding WRKY transcription factor 70; and *LOC104119049*, encoding probable WRKY transcription factor 41. We also used qRT-PCR to detect the expression levels of the above downregulated genes in the stem of transgenic tobacco (Figure 5E). These findings suggest that *LkABCG40* is possibly involved in the environmental adaptation and resistance of transgenic tobacco.

## 3. Discussion

In our study, we investigated the transcriptomic changes in the stem tissues of transgenic tobacco plants overexpressing *LkABCG36* and *LkABCG40* and compared them with those in the wild type. Our findings showed that the functions of the DEGs in the stem tissues of transgenic tobacco overexpressing *LkABCG36* were mainly enriched in plant hormone signal transduction, carbohydrate metabolism, and the biosynthesis of other secondary metabolites. The functions of the DEGs in the stem tissues of transgenic tobacco overexpressing *LkABCG40* were mainly enriched in metabolism, biosynthesis, and environmental adaptation. Based on the above results, we speculated that *LkABCG36* and *LkABCG40* play crucial roles in plant development and environmental adaptation.

AtABCG36 possibly plays a dual role in IBA transport [20]. The phytohormone auxin IBA is involved in regulating plant growth and development, and it plays an important role in the responses to biotic and abiotic stresses, including drought, salinity, and cold [23]. In this study, we found that transgenic tobacco overexpressing *LkABCG36* grew rapidly, which may have been related to *LkABCG36* catalyzing the export of IBA [20,21]. In addition, we detected many significantly upregulated genes encoding auxin or auxin receptors in the stem tissue of transgenic tobacco overexpressing *LkABCG36*; for example, *LOC109223084*, encoding auxin-responsive protein and *LOC104113066* and *LOC107784906,* encoding indole-3-acetic acid-amido (IAA) synthetase. This finding may be related to the dynamic spatiotemporal changes in the level of auxin that can accurately and rapidly trigger gene reprogramming [24]. Therefore, we speculated that the overexpression of *LkABCG36* promotes the expression of auxin response factor (ARF) and increases the secretion of auxin in transgenic tobacco. Furthermore, Yu et al. [25] found that IAA20 (*Eucalyptus grandis*) is preferentially expressed in cambium and is involved in wood formation. Zhang et al. [26] found that maize (*Zea mays* L.) Aux/IAA protein RUM1 plays key roles in the regulation of the auxin signal transduction components and promotes vascular development in primary roots. This indicates that Aux/IAA not only plays an important role in regulating plant growth and development, but also provides protection for plants in adapting to environmental changes [24]. Therefore, we hypothesized that *LkABCG36* promotes the expression of key auxin-related genes by transporting auxin, thereby accelerating plant growth and development, and ultimately enhancing the resistance of transgenic tobacco.

ABCG40 is also involved in hormone transport, but unlike AtABCG36 involvement in IBA transport, AtABCG40 is an exporter of abscisic acid (ABA) [10]. ABA is a stress hormone which plays key roles in regulating both biological and abiotic stress responses [27], and ABA improves drought tolerance and water-use efficiency by regulating stomatal conductance. Zhang et al. [28] found that AtABCG17 and AtABCG18 redundantly promote ABA import, restricting stomatal closure and thus allowing plants to respond to the changing environment. This suggests that ABCG40 may be involved in long-distance ABA transport, regulating the physiology and morphology of plants. In addition, we found that a number of the DEGs involved in environmental adaptation were significantly upregulated, such as *LOC104234183,* encoding probable disease resistance protein RF9; *LOC104215816*, encoding resistance protein homolog R1A-10; and *LOC104245491*, encoding resistance protein homolog R1B-16. These results indicated that the overexpression of *LkABCG40* promotes resistance in transgenic tobacco. In addition, we found some DEGs were downregulated in encoding WRKY transcription factor; examples include *LOC104245013*, *LOC104210912*, *LOC104115180,* and *LOC104213127*. The *WRKY* gene family is one of the largest transcription factor families in higher plants [29]. WRKY proteins encoded by *WRKY* genes often act as repressors and activators [30]. These transcription factors play important roles in the response to biotic and abiotic stresses, mainly by regulating plant hormone signal transduction pathways in plants [31]. The expression of WRKY is significantly reduced by drought stress or treatment with mannitol and ABA [32,33]. Furthermore, it has been found that the concentration of ABA is lower than that of WT in *Arabidopsis thaliana* with ABCG40 knockout [34]. Therefore, we hypothesized that in overexpression of *LkABCG40* the ABA concentration may be higher than that in WT, and the high concentration of ABA may inhibit the expression of *WRKY*.

However, some aspects need to be further explored, such as the difference in hormone transport between *LkABCG36* and *LkABCG40*, and whether they differ in their specificities for transport substrates. In addition, auxin is a growth hormone, and ABA is often defined as a stress hormone. Both of them play important roles in plant development and enhancing plant traits, but the complex cross-talk relationships between different plant hormones are not well understood, so the *LkABCG36* and *LkABCG40* hormone transport mechanisms may provide insight into the environmental adaptation of plants.

In conclusion, we found that *LkABCG36* and *LkABCG40* play important roles in plant development and environmental adaptation. *LkABCG36* may promote the growth of transgenic tobacco by promoting the transport of auxins such as IBA and IAA; *LkABCG40* may inhibit the expression of *WRKY* by increasing the concentration of ABA to improve the stress resistance of transgenic tobacco. These results are beneficial information for future studies into the functions of *LkABCG36* and *LkABCG40*.

## 4. Materials and Methods

### 4.1. Plant Materials

We sterilized tobacco (*Nicotiana tabacum*) seeds with 70% ethanol, then rinsed them three times with sterile water. After removing the disinfectant, we germinated the seeds on MS medium and placed them under a 16/8 h (light/dark) photoperiod at 25 °C for culture. To obtain *LkABCG36* and *LkABCG40* transgenic tobacco plants, we used *Agrobacterium* mediated transformation as previously described [35,36]. We collected leaves from 5-week-old tobacco plants, which we cleaned with reverse osmosis deionized (RODI) water, then disinfected with 4% NaClO. Following this, we washed the leaves three times with RODI water. Then, we cut the leaves into 1–2 cm^2^ explants, which we placed on a shoot-induction medium (SIM) for culture with the following supplements: 3% (*w*/*v*) sucrose, 1 mg/L 6-benzylaminopurine (BA), 0.1 mg/L 1-naphthaleneacetic acid (NAA), and 8% agar with pH adjusted to 5.7. In the next step, we immersed the explants in *Agrobacterium* (carrying a helper plasmid *pBI121-GFP*) suspension for 15–20 min. The sequences of *LkABCG36* and *LkABCG40* are shown in Supplementary Data S1.

We used sterile filter paper to blot the remaining fluid from the explants, which we then transferred to fresh SIM for co-culture over 24 h. Finally, we transferred the explants to a fresh SIM medium containing 30 mg/L meropenem and 200 mg/L kanamycin. When the roots of the explants reached 1–2 cm, we cut them into sections and transferred them into fresh RIM, which contained 3% (*w*/*v*) sucrose, 0.1 mg/L NAA, 30 mg/L meropenem, 200 mg/L kanamycin, and 8% agar with pH adjusted to 5.7, for further development. When the plantlets reached 5–8 cm in height, we transferred them to soil for acclimation and identification of transgenic positive plants. Ultimately, we obtained a total of 11 transgenic lines, and we selected a homozygous strain with high expressions of *LkABCG36* or *LkABCG40* for this study.

### 4.2. RNA-Seq and Data Processing

We derived the materials used for RNA-seq analysis from the stems of 11-week-old transgenic and wild-type plants, and we collected 3 samples from each group. We first extracted total RNA from the stem tissues using a Plant RN38 Kit (Aidlab, Beijing, China) according to the manufacturer’s instructions. Then, we prepared the RNA-seq library with a TruSeq RNA sample preparation kit (Illumina, San Diego, CA, USA), according to the manufacturer’s instructions; we sequenced the libraries on an Illumina HiSeq system. We used Skewer (v0.2.2) software (https://sourceforge.net) to dynamically remove joint sequence and low-quality fragments from the 3’ end of the sequencing data. We used FastQC (v0.11.5) software (http://www.bioinformatics) to conduct quality control analysis on the preprocessed data. We mapped the sequence data to the *N. tabacum* genome (https://solgenomics.net/organism/Nicotiana_tabacum/genome) using TopHat (2.0.12) software. We used StringTie (v1.3.1c) software (http://ccb.jhu.edu) to calculate the original sequence count of known genes for all samples, and we calculated the expression level of genes based on fragments per kilobase of transcript per million fragments mapped (FPKM) using Cufflinks software [37].

### 4.3. Differential Gene Expression Analysis

We analyzed the differentially expressed genes (DEGs) between WT and transgenic plants to identify significantly up- or downregulated genes. We assessed the DEGs using DESeq 2 (v1.16.1) software (https://bioconductor.org). We used the Benjamini–Hochberg multiple test correction method. We chose the DEGs according to |log_2_ fold change| ≥ 1 and adjusted *p* value < 0.05. We tested Gene Ontology (GO) and Kyoto Encyclopedia of Genes and Genomes (KEGG) (http://www.genome.jp) annotations for enrichment [38]. We constructed the gene co-expression network using weighted gene co-expression network analysis (WGCNA) methodology [39]. We plotted the heatmaps using Bioinformatics (https://www.bioinformatics.com.cn).

### 4.4. qRT-PCR Analysis

We selected a number of key genes for verification using qRT-PCR. We designed the primers with Primer Premier 5 software; all primers are listed in Table S1. We isolated the total RNA of the stems with a Plant RN38 Kit (Aidlab, Beijing, China), according to the manufacturer’s instructions. Then, we reverse-transcribed the total RNA into cDNA using a reverse-transcription kit (Aidlab, Beijing, China). We performed qRT-PCR using a 7500 Fast Real-Time PCR system (Applied Biosystems, Foster, CA, USA) with SYBR Premix Ex Taq^TM^ (Aidlab, Beijing, China), and we used *HSC70-1* as an internal reference gene. We ran the qRT-PCR program according to the manufacturer’s instructions, with three replicates for each sample. We used the 2^−ΔΔCT^ approach to assess the relative expression levels of key genes; data are reported as mean ± SEM (n = 4); and we analyzed the significant differences with GraphPad Prism 8.0 software with a *t*-test (* *p* < 0.05, ** *p* < 0.01).

## Data Availability

Not applicable.

## Supplementary Materials

The following supporting information can be downloaded at: https://www.mdpi.com/article/10.3390/plants12020227/s1, Table S1: The list of primer sequence.
