# Supplementary material for: Transcriptomic Insights into Functions of LkABCG36 and LkABCG40 in Nicotiana tabacum
Source: Plants (Basel). 2023 Jan 4;12(2):227. doi: 10.3390/plants12020227 (PMC9860546; doi:10.3390/plants12020227)
Supplement: Supplementary file 1 [file plants-12-00227-s001.zip › Date S1.pdf]

>LkABCG36

ATGATGAGGGGGTCTGGGGAGATGAGGAGTGGCAGTATGAGGAGTGGCAGGTTGAGTGGCAG
GATGAGTAACAGTATGAGGAGTGGTAGCAGATCCAGTTGGGGTTTTGAAGATGTGTTTGCAAAA
TCGGCAAGCACTCGGTCACAGAGAGAGAGAGTTGAAGATGACGAAGAAGCTCTCAGATTTGCA
GCTCTGGAAAAGTTGCCCACCTACGATAGGTTGAGGACCAGTATCATGCAAACGGTTCAGGAG
AGTGGTAACATGAACGTGGACTACAGACAAGTAAACGTTCGGAAGTTCGGCTTCACGGAAAGG
CAGATGTTCCTTGATCGTCTCATTCAGGTGGCAGATGAAGATAACGAGAAGTTCATGAGGACTC
TCAGGAATCGTATTGACAGAGTTGGTATTCAGCTTCCAACTGTAGAAGTCCGATTCCAACACTTG
ACAATCGATGCAGATTGTTCCGTGGGTGCCAGCGCTCTTCCTACACTATGGAATAGTGTAACAA
AACTGGGCGAGTACACGTTGGGCATGGCTGGATTGTCACTGACCAAGAAAACAAATCTCACAA
TTCTGAAAGACGCAAGCGGGATCATCAAACCATCTCGAATGACTTTGTTATTGGGTCCCCCTGG
GTCTGGAAAAACAACATTGCTGCTGGCTTTGGCTGGAAAATTGGACACTGGCTTGAAGGTGAGG
GGAGAGATTACTTACAACGGGCATACACTCCACGAATTTGTTCCTCAGAAAACATCTGCTTACA
TAAGCCAACGTGACTTACATGTTGGAGAAATGACAGTTAGAGAAACACTTGATTTCGCTGCTAG
ATGTCAAGGTGTTGGAGATAGATTCGAGCTGCTTACCGAGCTCGCTAGGAGGGAGAAGCAAGC
TGGGATTTTTCCAGAGGCAGACGTTGATCTATTCATGAAAGCAACAGCAATGGAAGGTATAAAG
AGTAGCCTTCAAACTGACTACATAATGAAGATTCTGGGCTTAGATGTTTGTGGAGATACAATAGT
TGGCAATGAGATGCAAAGAGGAATCTCCGGTGGGCAAAAGAAGCGTGTTACAACTGGGGAGA
TGACTGTTGGACCTACAAAGACTCTCTTCATGGATGAGATATCAACAGGGCTGGACAGTTCTAC
CACCTATCAGATAGTGAAATGCCTGCGGCAGTTCACTCATTTCATGGATACAACAATTTTCATGT
CTCTGTTGCAGCCAGCACCAGAAGTATTTGAGCTCTTTGATGAGATAATATTGTTATCGGAGGGT
CAAATTGTCTACCATGGGCCTAGGGAGAATGTCCTGGAGTTTTTTGAAAGCTGTGGATTCAAAT
GCCCTGAAAGAAAGGGCACTGCAGATTTCTTACAAGAGGTTACATCAAAGAAGGATCAAGAAC
AATACTGGGAAGACAAGGGGCAGCCTTATCGCTACATACCTGTCAAGGAATTTGTATTTAGATT
CAAAGAGTTTCATGCAGGTTTGGATTTGGAAAATGAGCTTTCAATCCCTTATGAGAAGGAGAGA
AGTCACAGAGCAGCTTTGGTGTTCGATAGGAACACTGTTCCCCCAATGGAGATCTTCAAAACAT
GTTTCGCTAAAGAATTGCTTCTGATCAAGCGAAATTCCTTTGTTTACATCTTTAAGACAGCACAG
ATTGTGGTTGGAGCCTTTATTGCATCGACTGTAATCATCAGGACTGAAATGCATCAAAAGACCAT
AAGTGATGCAAATGCATACCTCGGAGCACTTTACTACAGTCTCCTCACCAACATGTTCAATGGAT
TTGCAGAATTGGCCATGACAGTTGTCAGGCTCCCTGTGTTCTTCAAGCAACGGGATTTACTCTTC
TATCCTGCATGGGCTTTCACTATTCCTACTGTTTTACTGGGAATTCCATTGTCATTGTTGGAGTCT
GGGGTTTGGGTAATAATAACTTACTACACCATTGGGTTTGCCCCTGAAGCTAGCAGATTCTTTCG
ACAATTCCTTATTATCTTCCTAGTTCATCAGATGGCGTCGTCCTTGCTCCGTCTTATGGCAGGAGT

TTTTAGGTCAATGATATTAGCTAATACTGGCGGCTTTTTAGCTCTTCTGCTTGTAGTCATTCTTGG

AGGATTCATTTTACCAAGAGATAACATCCCAAAATGGTGGATATGGGGCTACTGGTGTTCCCCTT

TGATGTACGCAGAGAATGCGATTGCCGTGAACGAGATGCTTGCTCCCAGATGGATGATAAGTG

CAGATAATACCGAAAACTTGGGTGTTTCTGCGCTGGAGAGTCATGGCATCTTTACAAAAGAATA

TTGGTATTGGCTAGGAGCGGGCGCTTTGTTGGGATTTTCAGTACTCTTCAATGTGCTTTTTACGTT

GGCTCTTCAATATCTAAATCCTCTTGGGAATCCTCAAGCTGTCATTTCAGAAGAAGCTTTTAAAG

AAATGCAAGCCCAAGAAAATGATACAGAACAAGGGCATAATAGGAAGCCATCGAGATCGAGA

CGAGGGTCTTCTTCAGATGACAATAATACCCTCGAAATGCAGATGAGAAGAACGAACAACCAA

TCAATTAGTATGGAAGGCAATAATGAGATGGCTTTGAACGCTGCAACAGGAGTTCTTCCAAAAC

GAGGAATGATTCTTCCATTTCAACCACTAGCCATGTCTTTTGAAGATGTCAATTACTTCGTTGACA

TGCCCCCTGAATTAAGGGAACAAGTCGCTGAAAATAGACTTCAACTGCTCCAAGATGTTACAGG

GGCATTCAGACCTGGTGTTCTTACAGCATTGATGGGGGTCAGTGGTGCTGGGAAGACAACTCTA

ATGGACGTCTTAGCTGGAAGAAAGACTGGAGGATACATTGAGGGTGATATCAAAATCTCTGGAT

TTTCCAAAAAGCAAGAGACCTTTACTCGGATTTCCGGTTACTGTGAACAGAATGATATACATTCT

CCACAAGTAACTGTCCGGGAATCCTTGTTATATTCAGCATTTCTCCGTCTTCCAAACGATGTTGA

CAATGAAACCAAAATCGCCTTTGCTGATGAGGTCATGGAACTTGTGGAATTAGATAATTTAAGT

GATGCACTGGTAGGTTTGCCTGGTGTGACTGGATTGTCAACCGAACAAAGGAAAAGGTTAACA

ATAGCTGTCGAGCTTGTTGCAAATCCTTCTATAATTTTCATGGATGAGCCGACTTCTGGTTTGGAT

GCTAGAGCAGCTGCGATTGTGATGCGAACTGTTCGTAATACAGTAGATACTGGGAGGACTGTTG

TCTGTACGATTCATCAGCCTAGCATTGACATCTTTGAAGCTTTTGATGAGCTCTTGTTAATGAAAA

GAGGAGGGAAAGCCATTTTCTTCGGGCCACTGGGCCAGAATTCTCACAAGCTTGTCGAATACTT

TGAGGCCATTCCAGGAGTACCAAAGATCAAAGATAAATACAACCCAGCTACATGGATGCTGGA

AGTTAGTTCTATTGCTGCTGAACATGGTTTGGGGATAGACTTTGCAGAGTATTACAAGAACTCTT

CCCTCTATAAGAAGAACAGAGCATTGGTGAAACAGCTTAACATTCCACCTCCAGGATCGACGG

ATCTTTATTTTGCAACCCAGTACTCACAATCATTCTTTGGACAGTTCAGTTCCTGCTTGTGGAAGC

AATGGTTGACTTACTGGCGGAGTCCTGATTATAACATTGTTAGATACACATTCACATTTGTGTGT

GCTCTAGTGCTGGGAACAATCTTTTGGAAGGTTGGCAAAAAACGGGACAACAGCAATGATATTT

TTACTGTTGCGGGGGCCTTGTTTAGTGCAGTTATATTCTTAGGAGTTAACAACTGTTCAACTGTG

CAGCCTGTGGTTGCAGTAGAGAGAACTGTGTTCTACAGAGAAAGGGCTGCAGGGATGTACTCT

GCATTGCCCTATGCTCTGGCTCAGGTGGTGATTGAGCTTCCATACGTACTCGGCCAAACAGCTA

TGTACACTCTCATTGTCTATAGTATGTTCCATTTCGAGTGGACAACTAAGAAGTTTGGCTGGTTCT

TCTTCGTGAACTACTTCACATTTCTCTACTTCACCTATTATGGGATGATGACACTTTCCATCACTC

CAAACCAACAAGTGGCCTCAATAGTGGCTGCAGGATTCTACTCTATATTCTGTCTGTTTTCAGGA

TTCTTCATTCCCAAATCAAAAATCCCCAAATGGTGGATCTGGTACTACTGGATATGCCCAGTTGC
CTGGACAATGTATGGTCTCCTTGCCTCGCAATATGGGGACATTGCAGCTTCTCTGGATGTAGTG
GGTGGTGAAAAGGAATCACTGAGTAAGTTCGTGGAGGTTTACTATGGATACAGTTATGATTTCTT
GGGTGCAGTAGCTGGAGCCCTGGTCGGATTCTGTGTTCTCTTTGCGTTTGTCTTTGCTTACGGCA
TCAAGGTCCTGAACTTCCAACAAAGGTAA

>LkABCG40
ATGAGTTCGTCCACCGAGCATGAGCCTGATGATAAAACATGGACAGGCAGTCTGCGTTCAGCG
AGTACCAGATTGTTCGAACGATCCGACAGCGTTTTTTCTAAATCATCCAGTACTCGACAACGCCA
GAATGACGAGGAAGCGCTCAAATGGGCGGCCATTGAAAAGCTCCCCACTTACGACCGGTTAAG
AACTGCGATTCTTGCAAATGTACATGACAAAGGGGATATCGATCACAATCAGATTGACGTTCGG
GGTATCGAACTCGAGACCCGCCAACAGTTGATGGAGAGGCTCGTCAAAGTTGCAGACGAGGAT
AATGAACGCTTTCTTAACAAGCTTCGAAAGAGGATTGACAGTGTTGGCATCGTTTTGCCAGAGA
TCGAAGTTCGCTACGAACATTTGAACATCGATGCGAATGCCTACGTAGGTGGCAGAGCGCTGC
CCACTCTTACCAACTACACCGTCAACATGATCGAGGCAGTTCTTGGTTCGCTTCATTTGTACAAG
AGCAACAAGGCAACCATGACAATTCTCCATGATGTCAGTGGCATCATCAAGCCCGGCAGGATG
ACCCTTCTCCTTGGACCGCCAGCCTCGGGCAAGACTACTTTGCTCATGGCTCTTGCGGGAAAAC
TGGAGAAAAGTCTCAAAGTTACAGGTGGAGTAAAATACAATGGACATACCATGGATGAATTCGT
GCCACAGAGAACATCCGCTTACATAAGTCAAAATGACTTGCACATTGGTGAAATGACCGTGAGA
GAAACCTTAGATTTCTCTGCACGGTGCCAGGGCGTCGGGTCGAGATATGACGTATTGACTGAAC
TTGTGAGGAGAGAAAAGGATGGAGGAATCAAACCAGATCCCGATATAGATGTCTTTATGAAGG
CCACTGCATTAGAGGGCCAGAAGGCCAACATGATTACGGACTATGTTTTGAAGATCTTGGGGCT
GGACATATGCGCCGACACACTAGTAGGAGATGCAATGCATAGAGGCATTTCTGGAGGGCAGAA
GAAAAGAGTTACAACTGGGGAAATGATTGTTGGAGCTTCGAAAGCCCTGTTCATGGACGAGATT
TCCACTGGTCTGGACAGCGCTACAACATACCAGATTGTTCGATGCCTACGCCAAGTTGTCCATG
TTTTCATGTCGACGATGGTGGTTTCACTCCTGCAACCTGCCCCTGAAACCTTCGATCTCTTCGAC
GATGTCATTCTACTTTCGGAAGGTCACATTATTTATCAGGGTCAGCGAGAAAACATTCTCGAGTT
CTTCGAGTCCATGGGATTTAAATGCCCTGAGAGGAAAGGTGTCGCAGATTTCTTGCAGGAGGTG
ACTTCTCTCAAAGATCAAGAGCAGTACTGGGCAAACAGAAGGCAAACGTACCGTTATGTTCCAG
TGAAGGAGTTTGCAGATGCATTCCAGTCATTTCACATTGGTACCGCATTGAATTCAGAGTTATCT
ATTCCATACGATAAGAAGAAATCCCATCCCGCGGCTCTGACAACAGAGGAATACGGTTTGAGC
AAAATGGAACTTTTCAGAGCTTGCTTTGATAGAGAAGTTTTGCTCATGAAGAGAAGTTCGTTTGT
CTACATCTTCAAAACTGTTCAGATATTTGTCATTGCTTCTATTGCCATGAGCGTCTTTTTCCGTACC

AATATGCATCACAGAAATCTGGGCGACGGAGGCATTTACTTTGGAGCGCTTTTCTTTGGTCTAGT
GGTGGTGATGTTCAATGGCATGGCAGAATTGTCGATGACAATTGACAAGCTTCCAGTGTTTTAC
AAGCAAAGAGATTTCAAGTTCTACCCAGCCTGGGCTTATTCGCTTCCGACATGGATTATGAGAA
TTCCTCTTTCCCTGTTGGAGTCTGTGCTGTGGATCATTATCACATATTATACCATAGGTTTTGCCC
CAAATCCTCAGAGATTTTTCCGACAACTTGTTCTGTTCTTCACAGTCCATCAGATGTCTTTAGGTC
TTTTTCGCTTTATAGCTGCTGTGGGACGCAATCGGATAGTGGCTAACACTTTCGGTTCGTTTGCC
CTCTTGATCATACTCGTTCTCGGAGGATTCATCATAACCAGAGATGATATTAAAGGATGGTGGAT
ATGGGGCTACTGGATTTCTCCTCTCATGTATGCTCAAAATGGAATTGCAGTAAACGAGTTCTTGG
CAAGCAGATGGCAAAAGCCAGGTGATAACGTTGGCGTCAATTTATTGGAATCTCGCGGGCTTTT
TGCGAAGAGCTACTGGTATTGGCTTTCTATTGGAGCCATGGTGGGATTCAATATTTTATTTAACTT
CCTCTACACGTTGGCATTGCATTATCTAGATCCTCTAGGAAAGCCCCAGGCATTAATTTCTGAAG
ATGATTTGAAACAAAAGCAAGCAGCTCAAACCGGCGTTGTCCAAAGAAACAATACAAATGAAA
TGCAGACGATGTCCGGAGAAAGCCATGAAGAAATTCAAGCTTCTGATTCTGTCAGGGCAAGTTC
ACGGGCAAGTTCACAACGAGGACGAAATAGCAATATTTCAGATTCTATTCGTGCATCTGGAAAG
AAAGGAATGGTTTTACCTTTTCAACCTTTGGCGATGGCCTTTGACAATGTCAATTATTTTGTAGAC
ATGCCCCCGGAAATGAAGCAGCAAGGAGTTGAAGAAAGTAGACTCCAGTTATTGCGTGGAACG
AGTGGGACATTCAGGCCTGGCGTGCTCACCTGTTTAATGGGTGTGAGTGGGGCTGGAAAAACA
ACCCTAATGGATGTTCTTGCAGGAAGGAAAACTGGTGGATACATAGAAGGGTCTATCACAATCT
CCGGTTATCCCAAGAAACAAGAAACCTTTGCTCGCATCTCTGGTTACTGCGAACAAACTGATAT
CCATTCTCCTTTTGTTACTGTTTATGAGTCTCTCGTATACTCTGCCTGGCTTCGTCTTCCGGCCGA
AGTCGATACTGAGTTAAGAAAGACGTTTGTGGAGGAAGTTATGGATCTTGTGGAGCTGAATAAC
CTGAGACAAGCCCTGGTCGGTCTTCCTGGAGTGAATGGCCTGTCCACGGAACAACGTAAGAGA
TTGACAATAGCAGTTGAATTGGTTGCAAATCCTTCAATAATCTTCATGGACGAGCCAACATCAG
GACTGGATGCGAGAGCTGCTGCTATTGTGATGAGGACTGTCCGAAACACTGTGGATACAGGCC
GAACTGTTGTGTGCACTATTCATCAACCCAGCATTGATATCTTCGAGGCTTTTGATGAGCTTGTG
TTGATGAAGCGAGGAGGACAGATGATATACGTTGGTCCTCTTGGCCGCCATTCCCAATCACTGA
TCGACTACTTTGAGGCCATTCCAGGCGTTCCCAAAATCACCCATGGATACAACCCTGCAACTTG
GATGCTTGAAGCGTCTTCTGTTGGCGCAGAATTGCGTCTTGGAGTCGATTTTGCAGAAGTATACA
GGAATTCATCCCTCTACCAGCGAAATGAAGCTCTGATCAAGGAACTCGGCGTACCGGCTCCAG
GAACTAAAGATATCTACTTCGAAACCGAATTTTCACAATCCTTTGCAATTCAGTCCTTGGCCTGTT
TGTGGAAGCAGCACTGGTCATACTGGAGAAATCCCGAGTACAATGCTGTCCGTTTCTTTTTCACT
CTGGTCACTGCTGTGCTTTTCGGCACCATCTTTTGGAGAATGGGTCAAAAGATAACGGAGCAAC
AAAAGGTTTTGAATGCTATGGGGTCGATGTTTGCGGCGGTGCTATTCATTGGTGTGAACAATGCT

TCTTCAGTCCAGCCTGTGGTGGATGTTGAAAGGACAGTTTTCTACAGAGAAAAGGCAGCAGGG

ATGTACTCGGCCCTTCCTTATGCCCTCGCTCAGGTACTGATCGAAGTGCCATACGTCTTTGTTCA

AGCGGCGGTGTATGGAGTAATCGTGTATGCAATGATCGACTTCGAGTGGAAGGCAACCAAGTT

CCTGTGGTTTTTCTTCTTCATGTTCTTTACATTTTTGTACTACACATACTATGGAATGATGACGGTA

GCTCTGACTCCCAATGTGAACATTGCTGCCATTGTGTCCTCGGCTTTCTATTCCATCTGGATGCTT

TTCTGCGGTTTCATAATTCCTCGACCGAAAATTCCGGTATGGTGGAGATGGTATTACTGGGCATG

CCCTGTTGCATGGACTCTGTACGGTTTGATAGCATCGCAGTTCGGGGATTATGATGGTCTCATGA

CTAGAACAGACGGAACTAAACAGCCATTAAATGAGTTTATTAGAAGTTATTTTGGATTCAAACAT

AGCTTTTTGGGACCGGTTGCAGTTATGACAGCAGGATGGAGCGTTCTCTTCGCATTCATCTTCGC

ATTCTCCATCAAGAAGCTCAATTTCCAGTCAAGATAA
